# A Model Based Cost-Effectiveness Analysis of Routine Genotyping for *CYP2D6* among Older, Depressed Inpatients Starting Nortriptyline Pharmacotherapy

**DOI:** 10.1371/journal.pone.0169065

**Published:** 2016-12-29

**Authors:** Elizabeth J. J. Berm, Judith J. Gout-Zwart, Jos Luttjeboer, Bob Wilffert, Maarten J. Postma

**Affiliations:** 1 University of Groningen, Groningen Institute of Pharmacy, Unit of PharmacoTherapy, -Epidemiology and -Economics (PTE2), Groningen, the Netherlands; 2 University of Groningen, University Medical Center Groningen, Department of Clinical Pharmacy and Pharmacology, Groningen, the Netherlands; 3 University Medical Center Groningen (UMCG), Institute for Science in Healthy Aging & HealthcaRE (SHARE), Groningen, the Netherlands; 4 University Medical Center Groningen (UMCG), Department of Epidemiology, Groningen, the Netherlands; University of Miami School of Medicine, UNITED STATES

## Abstract

**Objective:**

Genotyping for *CYP2D6* has the potential to predict differences in metabolism of nortriptyline. This information could optimize pharmacotherapy. We determined the costs and effects of routine genotyping for old aged Dutch depressed inpatients.

**Methods:**

With a decision-tree, we modelled the first 12 weeks of nortriptyline therapy. Direct costs of genotyping, hospitalization, therapeutic drug monitoring and drugs were included. Based on genotype, patients could be correctly, sub-, or supratherapeutically dosed. Improvement from sub- or supratherapeutically dosed patients to correctly dosed patients was simulated, assuming that genotyping would prevent under- or overdosing of patients. In the base case, this improvement was assumed to be 35%. A probabilistic sensitivity analysis (PSA) was performed to determine uncertainty around the incremental cost-effectiveness ratio (ICER).

**Results:**

In the base case analysis, costs for genotyping were assumed €200 per test with a corresponding ICER at €1 333 000 per QALY. To reach a €50 000 per QALY cut-off, genotyping costs should be decreased towards €40 per test. At genotyping test costs < €35 per test, genotyping was dominant. At test costs of €17 per test there was a 95% probability that genotyping was cost-effective at €50 000 per QALY.

**Conclusions:**

*CYP2D6* genotyping was not cost-effective at current genotyping costs at a €50 000 per QALY threshold, however at test costs below €40, genotyping could be costs-effective.

## Introduction

Major depressive disorder (MDD) is a disease with a significant burden of disease in the aging European population [1]. Antidepressants can be used in the treatment of MDD. There are different classes of antidepressants, with the tricyclic antidepressants (TCAs) representing a treatment option typically initiated after unsuccessful treatment with a selective serotonin reuptake inhibitor (SSRI) [2]. Based on the most favorable side-effect profile, nortriptyline is the TCA of first choice among older patients as advised by different guidelines [3,4]. In contrast to most SSRIs, TCAs display a clinically relevant concentration-effect relationship and therefore therapeutic drug monitoring (TDM) is strongly recommended [5].

Nortriptyline is metabolized by the polymorphic cytochrome P450 2D6 (*CYP2D6*) enzyme [6]. Generally, its polymorphisms are classified into four different phenotypes: poor metabolizer (PM), intermediate metabolizer (IM), extensive metabolizer (EM) or ultra-rapid metabolizer (UM) [7]. Notably, EMs represent the most prevalent group in Caucasian populations. Compared to the EMs, PMs and IMs have a decreased activity of the *CYP2D6* enzyme, whereas UMs have an increased activity of this enzyme. As a result of this variance in enzymatic activity, patients display large variations in plasma concentrations despite similar drug dosages [8–10].

In addition to TDM, to further improve pharmacotherapy with nortriptyline, routine testing for *CYP2D6* has been proposed to facilitate dose adaptations in an early stage of pharmacotherapy [11]. Especially in aged patients, genotyping might be beneficial, because patients above 60 years are more frequently exposed to higher plasma concentrations of nortriptyline [12]. Although the literature is not completely unambiguous, these higher plasma concentrations might relate to more and/or more severe adverse drug reactions (ADRs) [13,14]. Besides a reduction in ADRs, better efficacy of nortriptyline can be expected in patients whose plasma concentrations are within therapeutic range, since sub- or supratherapeutic plasma concentrations of nortriptyline can reduce the efficacy of the drug [5,15,16]. Potentially related to reductions in ADRs and an increased efficacy, suggestions have been made that *CYP2D6* genotyping can reduce psychiatric hospitalization costs [11,17–20]. For schizophrenic patients who used *CYP2D6* dependent antipsychotic agents, this was recently demonstrated in Denmark [21].

To facilitate dose finding of nortriptyline with the use of genetic information, specific guidelines for dose adaptations of nortriptyline have recently become available [22]. Indeed, in some secondary psychiatric care facilities in the Netherlands, genotyping is already implemented as care as usual [23]. However, no information concerning the cost-effectiveness of routine genotyping at the start of nortriptyline treatment among depressed hospitalized patients is available. We constructed a pharmacoeconomic model, to assess cost-effectiveness of routine genotyping. As safe and fast dose finding is considered particularly important among severely depressed aged patients, we designed the model to simulate a Dutch hospitalized population of 60 years and older.

## Methods

### Design

To evaluate the cost-effectiveness of *CYP2D6* genotyping, a decision-tree was built in Microsoft Excel version 2010. Within this model, two virtual cohorts of 1000 depressive patients with an age of 60 years or older and treated with nortriptyline were simulated. In one cohort patients were genotyped whereas in the other patients received care as usual. Subsequently, costs and health outcomes of both cohorts were compared.

**Fig 1:**
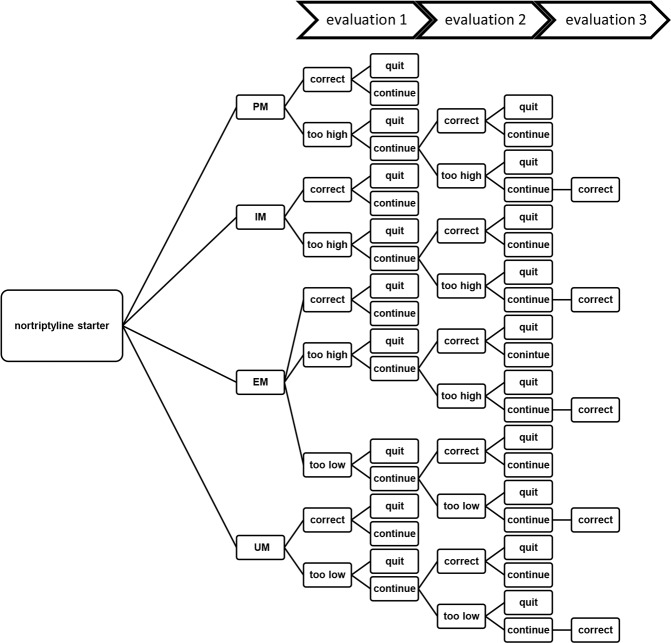
Model structure for the treatment of major depressive disorder with nortriptyline during the first 12 weeks. Patients who receive a dosage which led to therapeutic plasma concentrations (i.e. “correct”) did not receive a next dose evaluation. Patients who quit nortriptyline pharmacotherapy entered a wash out period with a disutility of 0.20 for 14 days. After this period, pharmacotherapy with 6 tranylcypromine was initiated for the remaining time in the model. PM = poor metabolizer, IM = intermediate metabolizer, EM = extensive metabolizer, UM = ultrarapid metabolizer.

According to the Dutch guideline on depression (addendum elderly), patients should be titrated towards a dose of 75 mg per day which takes approximately 12 days [4]. At this dose, plasma concentrations should be evaluated. Therefore, we assumed that in the model, plasma concentrations of all patients were evaluated after 12 days. As a result of this evaluation, patients were labeled to have either received a correct dosage, suboptimal dosage, supratherapeutic dosage or had discontinued therapy. Patients who were not optimally dosed received a dose adaptation and a second evaluation of plasma concentration. Patients who were still incorrectly dosed after this second evaluation received one more dose adaptation and control of plasma concentration and were assumed to be correctly dosed afterwards. Patients who discontinued nortriptyline pharmacotherapy entered the next step of treatment (i.e. tranylcypromine 40 mg) which was initiated after a wash out period of 14 days [2]. The structure of the decision-tree is shown in Fig 1. For both the “care as usual” and “genotyping” cohorts the same structure was used, although the input variables were different.

All patients in the simulated treatment cohorts were hospitalized in a psychiatric hospital for an episode of severe depression which was followed by discharge. Patients were structured by their genotypes. For simplification of the model, different genotypes led to specific assumptions. First, PMs and IMs could only receive a dose that was either correct or too high. In addition, after dose adjustment, dosing could not become incorrect in an opposite way. For example, when an EM was dosed too high, the dose could not become too low after adjustment. An UM could only receive a dose that was either correct or too low.

As mentioned before, most patients had an EM genotype, however distribution of genotypes is dependent on the ethnicity of a population. For this study, we used frequencies that are reported for European Caucasian populations (Table 1) [24,25].

**Table 1:** Main model inputs and variables of deterministic and probabilistic sensitivity analysis.

Variable	Base case	Range deterministic sensitivity analysis(min- max)	PSA distribution (parameters)	Source	Old age specific?
**Effect of genotyping (i.e. improvement of incorrectly dosed to correctly dosed) (%)**	35	31–38	β (253, 470)	[28]	No
***Incorrectly dosed until first evaluation (%)***					
**Poor metabolizer too high**	76	73–78	β (735, 239)	[28]	No
**Intermediate metabolizer too high**	56	53–59	β (572, 443)		
**Extensive metabolizer too high**	12	n.a.	n.a.		
**Extensive metabolizer too low**	25	n.a.	n.a.		
**Ultra-rapid metabolizer too low**	57	54–60	β (593, 455)		
***Incorrectly dosed until second evaluation (%)***					
**All genotypes**	43	34–53	β (40, 53)	S1 Fig	Yes
***Additional days after start at which an evaluation took place***					
**Dose evaluation 1**	12	5–19	γ (11, 1.06)	[4]	Yes
**Dose evaluation 2**	31	Dependents on evaluation 1	Dependent on evaluation 1	S1 Fig	Yes
**Dose evaluation 3**	38	Fixed	Fixed	S1 Fig	Yes
***Genotype (% of population)***					
**Poor metabolizer (2 dysfunctional alleles)**	8	6–10	Uniform	[24]	No
**Intermediate metabolizer (1 dysfunctional allele)**	11	9–13	Uniform		
**Extensive metabolizer**	79	Dependents on other genotypes	n.a.		
**Ultra-rapid metabolizer(duplication of the gene)**	2	1–2	Uniform		
***Hospitalization***					
**Average duration inpatient care (days)**	28.6	+/- 25%	γ (61, 0.47)	[19,38]	Yes
**Shorter hospital stay when correctly dosed (%)**	13	0–26	β (40, 53)	[19]	No
***Patients who discontinue therapy***					
**After first dose evaluation (%)**	22	12–33	β (11, 39)	[55]	No
**After second dose evaluation (%)**	8.5	2–17	β (3, 37)		
***Disutility’s***					
**If dosed to high**	0.04	0.01–0.12	PERT (0, 0.04, 0.13)	[36]	No
**If dosed to low**	0.20	0.12–0.28	β (19, 76)	[35]	
**If discontinue nortriptyline therapy**	0.20	0.12–0.28	β (19, 76)	assumed	

### Time horizon

It was assumed that *CYP2D6* genotype information would only optimize dose finding of nortriptyline during the titration phase. Therefore, effects were limited to the timespan of the titration phase. According to expert opinion, this phase is usually completed within three evaluations. As such, we chose a time horizon of maximally three evaluations. To assess the time it would take for three evaluations to occur, we used guideline recommendations and retrospective (2009–2014) anonymous TDM data from the Clinical Pharmacy of the Diaconessen Hospital Meppel/Hoogeveen, the Netherlands (unpublished results; see S1 Fig). First, based on the advices in guidelines, all patients received the first evaluation 12 days after start of therapy [4]. Second, based on the TDM data, a second or third evaluation took place after 31 and 38 days. For a total of three evaluations, this resulted in a 12 weeks’ time horizon of the model. In the model it was assumed that after this period, patients were either correctly dosed or had discontinued nortriptyline pharmacotherapy.

### Effects of genotyping

For economic evaluations of pharmacogenomics the analytical and clinical validity of the genetic test has to be defined [26]. The analytical validity (i.e. ability of the test to differentiate between carriers of different alleles) depends on the method which is used, but is in general known to be very high: >99% for CYP-enzymes [27]. In the model it was therefore assume that the analytical validity was 100%. The clinical validity of *CYP2D6* testing (i.e. the ability of the test to differentiate between clinical characteristics, like a PM or UM phenotype) depends on the number and type of variant alleles which are analyzed. In the model, genetic variation in the CYP2D6*2, *3, *4, *5, alleles, and duplications of the gene were used for the clinical effects. These effects were based on the prediction of the PM and UM phenotype among a European Caucasian population. In a pharmacokinetic model it has been reported that when *CYP2D6* phenotype predictions are based on genetic variations in these alleles 78.5% of PMs would have supratherapeutic nortriptyline plasma concentrations at a normal daily dose [28]. In the pharmacokinetic model dosing of nortriptyline was adapted based on these *CYP2D6* alleles As a result, a 51.1% instead of 78.5% of the patients had supratherapeutic plasma concentrations in the PM group. This corresponded to a reduction of ~35% (1- (51.1/78.5) = 0.35) of inadequately dosed patients [28]. This reduction was used in the base case of our model for the PM group in the genotyping cohort. For the UM group, an improvement from 60.2% to 39.3% in suboptimal dosed patients was reported which almost equals the effect found in the PM group (i.e. a 35% reduction in inadequately dosed patients). This effect was also included in the model. In the base case no effect for IMs or EMs were assumed. In a scenario analysis we did include effects for IMs (see sensitivity analysis).

Depending on their genotype, PMs and UMs in the ‘genotyping’ cohort received a different start dosage compared to patients in the ‘care as usual’ cohort. Dose adaptations were based on the advices given in the Dutch guidelines: 40% of standard dose for PMs and 160% of standard dose for UMs [29]. A detailed dosing schedule can be found in S1 Table.

### Outcome measures

Better efficacy as well as a reduction of ADRs can be expected in patients who have nortriptyline plasma concentrations within the therapeutic range as compared to outside [5]. Both affect quality of life. For ADRs, reduced utilities (ranging from: -0.12 to -0.01) among patients who experience ADRs of antidepressants have been reported [30]. For efficacy, a difference in utility ranging from 0.22 to 0.45 between untreated and treated depression has been described [30–34]. In addition, a difference in utilities between responders and non-responders to antidepressant of 0.20 has been reported [35]. In the model we included a disutility of 0.04 for experiencing an ADR when dosed supra-therapeutically [36]. The reported difference of 0.20 between non-responders and responders was used as a disutility for subtherapeutic dosed patients, assuming that patients who were dosed too low, would not respond optimally to nortriptyline pharmacotherapy. Among patients who discontinued nortriptyline pharmacotherapy, a similar disutility of 0.20 was included for the wash out period of 14 days, before the next treatment step with tranylcypromin was initiated. In the model, these disutility’s were linked to the proportions of patients with sub, or supratherapeutic dosages, or to a wash out period for each day patients were in a certain ‘health state’.

For the first evaluation we assumed correctly dosed patients were patients with a nortriptyline plasma concentration within the therapeutic range. Proportions of therapeutic, sub-, and supratherapeutic plasma concentrations were extracted from literature [28]. After the first weeks of treatment, the effects in terms of antidepressant response, start to improve patient’s utility [37]. At this stage, in addition to plasma concentration, drug dosing is adapted based on clinical efficacy and ADRs. Therefore, in the model, the proportion of patients who were incorrectly dosed at the second evaluation was not based on the plasma concentration, but on the proportion of patients who had a third TDM request in the clinical pharmacy. As such, it was assumed that patients who were controlled by TDM for a third time were not correctly dosed before (S1 Fig). Notably, the TDM data had no information about differences between genotypes. Therefore, conservatively, we assumed similar proportions of incorrectly dosed patients among all genotypes.

In addition to an improved quality of life, a reduction in hospitalization days was included in the model. Hospitalization of US patients with MDD and a decreased *CYP2D6* metabolic capacity was found to be 13% longer than the average (7.8 instead of 6.9 days) [19]. Among the Dutch population, average hospitalization duration for MDD (from 2007 to 2011) of patients aged 65 or older was 28.6 days [38]. To translate US-results into a Dutch setting, we assumed that based on genotype guided dosing, a proportionally similar reduction of 3.7 hospitalization days from the average (0.13*28.6 = 3.7) was achieved among eligible patients, by shifting them from suboptimal to therapeutically dosed. As a result, patients who were correctly dosed after the first evaluation were discharged from hospitalization after 24.9 days (28.6–3.7 = 24.9). The remaining patients (i.e. suboptimal dosed patients) were assumed to be hospitalized for 31.6 days to realize the weighted average of 28.6 days as registered. Main model inputs are shown in Table 1.

### Costs

Our evaluation was performed from a health-care insurance payers perspective and only direct medical costs were included in the model (Table 2). This perspective was chosen, due to a lack of data concerning effects of genotyping on indirect costs and on the relatively modest productivity loss to be expected in a population of 60 years and older. Costs and utilities were not discounted, since our model had a time horizon of 12 weeks. Costs were expressed in 2014 price levels. Drug costs were collected from the website of the Dutch National Health Care Institute [39] and costs for genotyping and TDM were obtained from the Dutch Healthcare Authority [40]. Hospitalization costs and costs for consultations with a psychiatrist were obtained from guideline prices listings and converted to costs in 2014 using the appropriate deflators [41,42]. Drug and TDM costs were included in hospitalization costs and calculated separately after patients were dismissed from hospital stay. Based on an average hospital stay of 28.6 days all patients were assumed to have a first dose evaluation during hospitalization which entailed no extra costs [38]. For a second or third evaluation additional costs for monitoring were counted. These costs included costs for TDM as well as ¼ of a psychiatric consult for interpretation of the nortriptyline plasma concentration and, if applicable, dose adaptations. Costs for genotyping of *CYP2D6* were based on costs for qualitative DNA amplification at €38 per amplification [22]. Assuming genotyping would involve testing for five amplifications, costs would be €190 per *CYP2D6* test. Note that test costs can be different between labs, due to different methods used for genotyping (i.e. TaqMan analysis, Roche AmpliChip CYP450, whole DNA sequencing). Furthermore, we assumed genotype information would be available before or at the moment of start of nortriptyline treatment.

**Table 2:** Drug costs included in the model.

Type of costs	Costs in 2014
**Genotyping (5 DNA amplifications)**	€ 188.20
**TDM (per measurement)**	€ 23.11
**Hospitalization (per day)**	€ 255.62
**Ambulant contact with psychiatrist**	€ 190.62
**Nortriptyline**	
**10 mg**	€ 0.08
**25mg**	€ 0.15
**50mg**	€ 0.29
**Tranylcypromin**	
**40 mg**	€ 0.96

### Cost-effectiveness

The incremental cost-effectiveness ratio (ICER) was calculated by comparing costs and quality adjusted life years (QALYs) between the two cohorts. In addition break even test costs were calculated.

### Deterministic, probabilistic and scenario sensitivity analysis

Input variables for the deterministic sensitivity analysis are shown in Table 1. The outcomes of the deterministic sensitivity analysis was reflected in Tornado diagrams.

Threshold analysis was performed on all variables to calculate break even test costs and maximal test costs to reach the €50 000 per QALY cut off [43]. For further assessment of the uncertainty, a probabilistic sensitivity analysis (PSA) was performed. The distributions and parameters of the variables are shown in Table 1. In addition, the probability of cost-effectiveness at €50 000 per QALY was calculated for various genotyping test costs [43].

Two scenario analysis were performed. First, a scenario in which the proportions of incorrectly dosed patients at the second evaluation did differ per genotype was included. For this differentiation, the same estimates as for the first evaluation were used (i.e. % incorrect evaluation 1 = % incorrect evaluation 2). In the second scenario analysis dose adaptations for IMs (60% of standard dose) were included [29]. No data was reported about supratherapeutically dosed IMs or clinical effects of genotyping in the pharmacokinetic model which was used for the PM and UM genotypes. Therefore, we took the average of the EM and PM group and as such 56% of the IMs was supratherapeutically dosed. Clinical effects of IM phenotype predictions is unknown, but we assumed an similar effect of 35% improvement among incorrectly dosed patients. With respect to clinical validity, It has been reported that 38% of *CYP2D6* IMs are false positive [44]. This means, these patients would be dosed too low when dose adaptations would be made based on genotype. Therefore, in this scenario analysis another branch was included in the decision tree for IM patients who were subtherapeutic dosed. As a result, 38% of the IM patients who were incorrectly dosed in the genotyping cohort, were dosed subtherapeutic instead of supratherapeutic. ICERs, QALY loss, break even test costs and test costs to reach the €50 000 per QALY were calculated for both scenarios.

## Results

### Base case

In the base case scenario an ICER of €1 333 000 per QALY was found. If test costs were €40, an ICER of €50 000 per QALY was found and when test costs were €35 per test, test costs equaled costs savings (i.e. break even). At lower costs, genotyping would become the dominant strategy. A summary of the model outcomes is shown in Table 3. The difference in QALY loss were small. Genotyping resulted in a QALY gain of 0.12. The majority of costs concerned hospitalization costs in the first weeks of treatment. In the genotyping cohort, costs for inpatient care and follow-up were lower. According to the model, genotyping could save €32 294 of inpatient care costs, €2 195 for TDM costs, and €244 of drug costs. However, due to genotyping costs, total costs in the genotyping cohort were higher.

**Table 3:** Outcomes of the model for 1000 patients per cohort during 12 weeks of pharmacotherapy in the base case scenario.

	Care as usual cohort	Genotyping cohort	Difference	ICER (€)	Max. test costs for €50 000/QALY (€)
**Base case**					
Patients with 24.9 days of inpatient care (correctly dosed)(n)*	483	447	36		
Days of inpatient care (mean)	28.60	28.47	- 0.13		
Patients who stopped therapy (n)	248	247	-1		
Costs of care (€)	7 374 826	7 340 091	- 34 734		
Costs *CYP2D6* genotyping (€)	0	188 200	188 200		
Total costs (€)	7 374 826	7 528 292	153 466		
QALY loss	4.57	4.46	0.12		
				1 333 148	40
**Scenario analysis; including genotype based differences for incorrectly dosed patients at evaluation 2**					
Total costs (€)	7 375 299	7 528 218	152 918		
QALY loss	4.50	4.34	0.15		
				999 622	43
**Scenario analysis; including dose adaptations for IMs**					
Total costs (€)	7 374 826	7 497 172	122 346		
QALY loss	4.57	4.52	0.05		
				2 380 626	68

* Suboptimal dosed patients were hospitalized for 31.5 days.

### Deterministic, probabilistic and scenario sensitivity analysis

**Fig 2:**
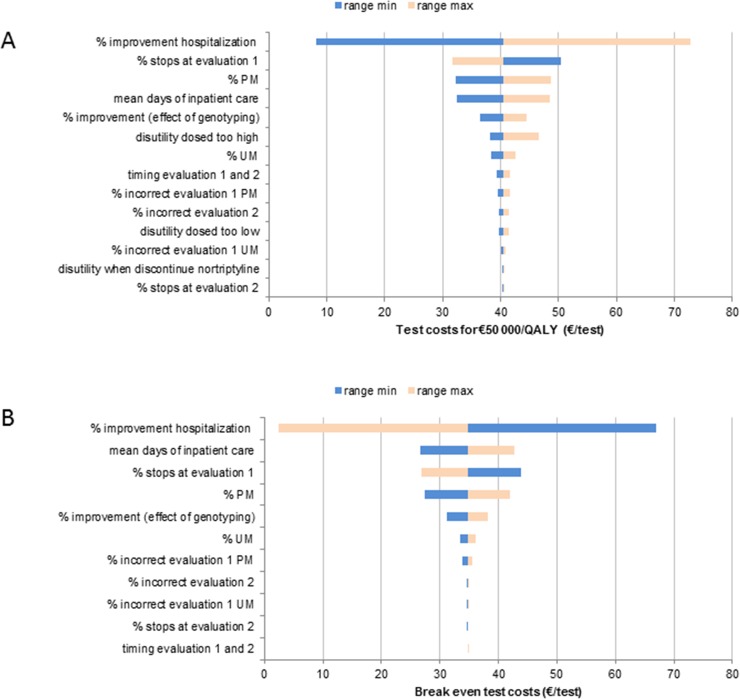
**Results of deterministic sensitivity analyses for genotyping test costs to reach the** €**50 000/QALY cut-off (A) or to break even with costs savings (B).**

The results of the deterministic sensitivity analyses are shown in Fig 2. Reduction in the duration of hospitalization among correctly dosed patients emerged as the most important parameter. When no reduction of inpatient care was assumed, test costs should be €2 to reach the €50 000 per QALY cut off, or €8 to break even. The proportion of patients discontinuing nortriptyline pharmacotherapy in the first weeks of therapy markedly influenced the ICER. When more patients continued nortriptyline pharmacotherapy, genotyping became more effective. With respect to quality of life, the utilities gained by preventing too high dosing had more influence on the ICER compared to the utilities gained by preventing patients who were dosed to low, even though there was more utility loss counted per case for the latter (0.20 vs. 0.04).

**Fig 3:**
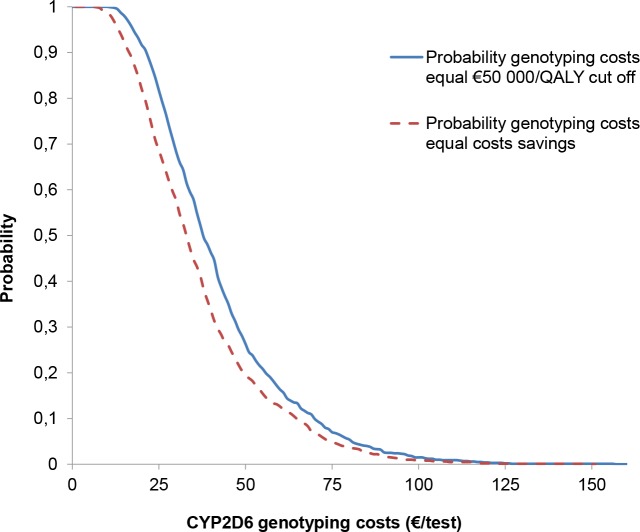
Probability on cost-effectiveness at €50 000 per QALY (solid line) or probability of test costs to break even with cost-savings (broken line) shown at different CYP2D6 genotyping test costs.

The PSA revealed that in the base case scenario (i.e. at current genotyping costs) a probability applied of <1% for genotyping to be cost-effective (€50 000 per QALY). If genotyping costs were €17 a probability of >95% genotyping to be cost-effective applied (€50 000 per QALY). Cost-effectiveness acceptability curves of the maximal *CYP2D6* test costs to obtain an ICER of €50 000 per QALY and breakeven test costs are shown in Fig 3.

The first scenario analysis indicated that when proportions of incorrectly dosed patients at the second evaluation equaled those of the first evaluation, this would increase the test costs to reach the €50 000 per QALY cut-off with €3 (i.e. €43 vs. €40 per test in the base case). This scenario analysis had no influence on break even test costs. In the scenario analysis which included dose adaptations for IM, test costs to reach the €50 000 per QALY cut-off or break even were identified at €68 or €66, respectively, due to more costs savings by reduced inpatient care of correctly dosed IMs. Although the costs in the genotyping cohort were lower in this scenario, a QALY loss among false positive IMs was introduced by genotyping. This was due to a larger utility loss when dosed sub- instead of supratherapeutic. As a result, incremental utility gain in the genotyping cohorts was reduced with 0.07 towards a remaining QALY gain of 0.05 in the genotyping cohort. Consequently, a higher ICER of €2 400 000 was found. If the proportion of falls positive IMs was decreased towards 26% (instead of 38%) an ICER, similar to the base case scenario was found.

## Discussion

Our cost-effectiveness study assessed possible economic implications of genotyping for *CYP2D6* among older hospitalized and depressed patients who start nortriptyline therapy. The results were mainly dependent on genotyping costs. At a price of €190 per test, we found genotyping for *CYP2D6* unlikely to be cost-effective. In the PSA, a probability of <1% was found at the €50 000 per QALY threshold. However, when genotyping costs were <€35 per test, genotyping was found to be dominant. Within countries costs for *CYP2D6* genotyping can be different between labs. For example, in the UK costs of £30 (€39) and £500 (€645) are reported whereas in US costs of $439 (€363) are found, respectively [45]. In these labs, the variant alleles studied in our model are usually included and amended with analysis of other alleles. Based on our model, purchasing these tests for routine genotyping of patients who start nortriptyline pharmacotherapy will unlikely be cost-effective. Furthermore, broader genotyping packages exist, for example including other genes like *CYP2C19* and *CYP2C9*. For such packages, even higher test costs of $914 (€1106) are reported [46]. Nevertheless, many patients aged > 60 years will receive polypharmacotherapy. For these patients, information about different genes might be beneficial and cost-effective due to multiple drug-gene interactions. Pharmacogenetic tests which asses these multi-drug and gene interaction can be key towards cost-effectiveness of genotyping, although the generalizability of these studies will be challenging [47,48]. Besides genotyping costs, the main parameters which influenced the ICER were the improvement in the duration of hospitalization among correctly dosed patients, mean duration of hospitalization, and the proportion of patients who discontinued nortriptyline pharmacotherapy. In scenario analysis, inclusion of dose adaptations for IMs was found to increase the costs savings, but decrease QALY gain, due to false positive IM genotypes which resulted in more subtherapeutic dosed patients. Based on this model, it was found that inclusion of dose adaptations for IMs does decrease the cost-effectiveness of genotyping. To obtain a similar ICER as in the base scenario a better correlation than currently reported, between genotype and phenotype of IMs, is needed.

Our analysis has some limitations. Firstly, the model was based on some assumptions of which the most important one was the genotyping-facilitated effect in 35% of patients transferring from sub- or supra-therapeutically dosed to therapeutically dosed. Except for the pharmacokinetic modeling study, a description of such estimates was not found [28]. It is important to obtain better estimates about the effects of genotyping to increase the reliability of the established cost-effectiveness of genotyping. In addition, we only included genotype specific variation in the estimate of the proportion of incorrectly dosed patients at the first evaluation and not at the second evaluation, due to a lack of data. In scenario analyses, inclusion of such a differentiation was found to have almost no influence on the breakeven test costs and only a minor increase in the maximum test costs for the €50 000 per QALY cut-off. Secondly, the relation between ADRs and high plasma concentrations of nortriptyline is maybe not as straightforward as in our model [49]. Nevertheless, among antidepressants, tricyclic antidepressants like nortriptyline have a well-accepted concentration-effect relationship and ADRs can give a lower quality of life [5,30]. Moreover, the upper limit of the therapeutic range is not solely there due to the occurrence of ADRs. Patients with a supratherapeutic dose often display non-response, irrespective of ADRs, and therefore it is important to maintain the upper limit of the therapeutic range [9]. Therefore, a disutility of 0.04 for patients with supratherapeutic plasma concentration as included in our model, was considered reasonable. Lastly, we assumed patients who were dosed to low would not be dosed too high after dosed adjustments, and vice versa for patients who were dosed to high. This is an oversimplification of the reality, however, considering the relative wide range of the therapeutic window of nortriptyline is it a fair assumption for most of the patients. In addition, when not applying this simplification, the effects of including other branches in the model, would have had little effect on our estimates due to the small number (n <50) of patients with deviating genotypes who were still incorrectly dosed at the second evaluation.

Similar approaches as sketched here for depressed patients can be found in other disease areas. In a trial based analysis of genotyping for thiopurine methyltransferase (*TMPT*) in combination with azathioprine treatment it was found that clinicians continued to give a low dosing although the genotype information suggested otherwise [50]. Therefore, we did not assume an effect of genotyping among the EMs, however if such an effect can be demonstrated, this will markedly influence the ICER in favor of the genotyping strategy.

There are over 100 alleles reported which influence the *CYP2D6* phenotype. The prevalence of the different alleles depends on the ethnicity of a population [24]. Consequently, the relation between genotypes and phenotypes can be different between populations when tested for the same set of alleles and is likely to increase when a test includes more alleles [51]. According to their methods, Jornil *et al*. only included variation explained by four alleles and duplications of the gene in their estimates [8]. In our model, we included testing and therefore costs for these alleles. However, testing for additional alleles is likely to improve the linkage between genotypes and phenotypes (i.e. clinical sensitivity and specificity of the test). Therefore the 35% reduction of incorrectly dosed patients which we included as the effect of genotyping might be a slight underestimation. Nevertheless, testing for more alleles will likely, but not necessarily, increase costs as and will need additional economic evaluation. Beside clear differences like costs, differences between countries and labs in methods for analyses of the *CYP2D6* gene and the resulting analytical and clinical accuracy should be considered when extrapolating our results to other regions or countries.

Two studies assessing clinical utility of genotyping for *CYP2C9* in combination with warfarin therapy found contradicting results. Consequently, the authors emphasized the importance of the moment in time of genotyping and effect measurement [52,53]. For warfarin, methods to correct for differences in metabolism between patients like TDM, gender, and age based algorithms are well developed. Therefore, effects of genotyping depend on existing monitoring methods and the study design. Moreover, effects would predominantly be observed in the first weeks of therapy. With our model, we tried to capture such little effects in the first weeks of nortriptyline pharmacotherapy which might be challenging to find in observational studies. We assumed an optimal situation in which genotype information was available before start of treatment. This is an essential assumption, because if a lab is not able to provide the information at this moment in time, effects of genotyping will be diluted and the ICER will increase. On the other hand, if preemptive genotyping is applied, genotype information may already be available when a patient is diagnosed with MDD. Furthermore preemptive genotyping can be organized with better efficiency when compared to ad-hoc genotyping, since multiple samples can be grouped together for laboratory analysis. This could include more genes than the CYP2D6 gene only and in addition lead to lower genotyping costs, leading to a lower ICER. The provided information could be used in different situations during a patients’ lifetime and should therefore not only be allocated to MDD specifically.

Matchar *et al*., analyzed the possible effect, but not cost-effectiveness of genotyping for *CYP2D6* when a selective serotonin reuptake inhibitors (SSRIs) was indicated [54]. They used fluoxetine as a model SSRI to be influenced by *CYP2D6* polymorphisms. Similar to our model, the authors had to make substantial assumptions. In addition, they used an expert opinion to predict the clinical effects of genotyping in relation to treatment response. As a consequence, one of their main recommendations was to conduct a prospective study to assess the real clinical effects of genotyping. The same holds true for our model, however our assumptions were based on a thorough pharmacokinetic study [28]. In addition we performed an extensive sensitivity analysis to give insights into the uncertainty. Therefore we consider our estimates valuable for decision makers.

Currently some of the authors are performing a prospective trial (the CYSCE trial) to evaluate outcomes of CYP2D6 genotyping among patients who will be initiated on nortriptyline therapy. Patient inclusion already started when we conducted this cost-effectiveness analysis, so we were unable to use these outcomes for the trial design. However the trial gave insight in current processes in psychiatry in The Netherlands, which was necessary to build a realistic cost-effectiveness model.

## Conclusions

A cost-effectiveness analysis on effects of genotyping for the *CYP2D6* enzyme in older Dutch inpatients treated with nortriptyline was performed. The effects of genotyping were estimated with a decision tree. At current costs, genotyping was not cost-effective. Our finding suggests genotyping would be cost-effective (i.e. €50 000 per QALY) when genotyping costs were decreased towards €40 per test. When genotyping costs were decreased towards €35 per test, genotyping was found to be the dominant treatment option.

## Supporting Information

S1 Fig Data used to identify time horizon. Based on retrospective (2009–2014) collected TDM data from the Clinical Pharmacy of the Diaconessen Hospital Meppel/Hoogeveen, the Netherlands, 100 out of 264 (38%) patients aged ≥60 years received TDM for the second time and 43 out of these 100 (43%) patients received TDM for a third time.
(PDF)

S1 Table Drug dosing schedules used to calculate differences in drug costs. Dose adjustments were based on guidelines from the Dutch pharmacogenetics working group.
(PDF)
