# Supplementary figures and images for: A Model Based Cost-Effectiveness Analysis of Routine Genotyping for CYP2D6 among Older, Depressed Inpatients Starting Nortriptyline Pharmacotherapy
Source: PLoS One. 2016 Dec 29;11(12):e0169065. doi: 10.1371/journal.pone.0169065 (PMC5199075; doi:10.1371/journal.pone.0169065)

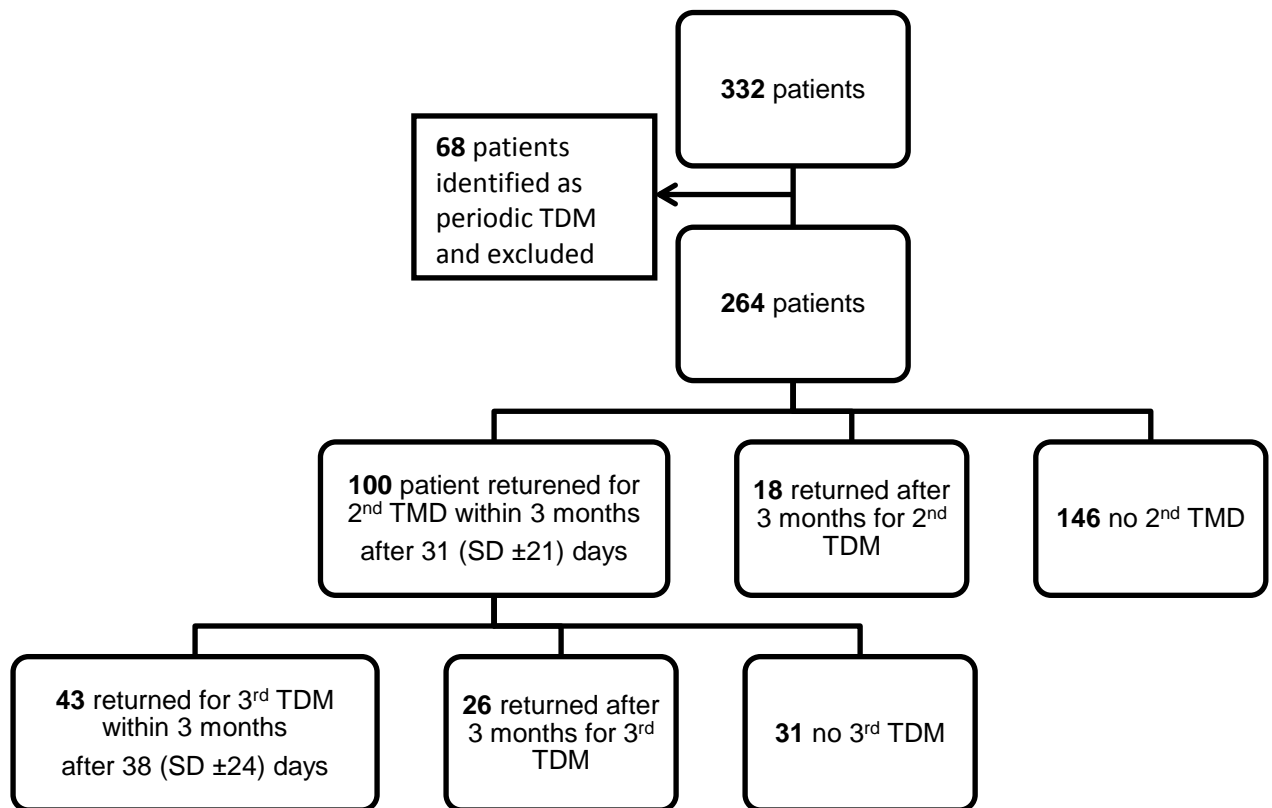

Supplement: S1 Fig — Based on retrospective (2009–2014) collected TDM data from the Clinical Pharmacy of the Diaconessen Hospital Meppel/Hoogeveen, the Netherlands, 100 out of 264 (38%) patients aged ≥60 years received TDM for the second time and 43 out of these 100 (43%) patients received TDM for a third time. (PDF) [file pone.0169065.s001.pdf]
