# Supplementary material for: A Model Based Cost-Effectiveness Analysis of Routine Genotyping for CYP2D6 among Older, Depressed Inpatients Starting Nortriptyline Pharmacotherapy
Source: PLoS One. 2016 Dec 29;11(12):e0169065. doi: 10.1371/journal.pone.0169065 (PMC5199075; doi:10.1371/journal.pone.0169065)
Supplement: S1 Table — Dose adjustments were based on guidelines from the Dutch pharmacogenetics working group. (PDF) [file pone.0169065.s002.pdf]

Care as usual cohort			
**Genotype**	Starting dose (mg per day)	Dose after first evaluation (mg per day)	Dose after second evaluation (mg per day)
**PM**	75	If too high: 50	If too high: 25
**IM**	75	If too high: 50	If too high: 25
**EM**	75	If too low: 100<br>If too high: 50	If too low: 125<br>If too high: 25
**UM**	75	If too low: 100	If too low: 125
**Genotyping cohort**			
**Genotype**	Starting dose (mg per day)	Dose after first evaluation (mg per day)	Dose after second evaluation (mg per day)
**PM**	30	If too high: 25	If too high: 10
**IM**	75	If too high:50	If too high: 25
**EM**	75	If too low: 100<br>If too high: 50	If too low: 125<br>If too high: 25
**UM**	125	If too low: 150	If too low: 175
**Genotyping cohort (as included in scenario analysis with dose adaptations for IMs)**			
**Genotype**	Starting dose (mg per day)	Dose after first evaluation (mg per day)	Dose after second evaluation (mg per day)
**PM**	30	If too high: 25	If too high: 10
**IM**	50	If too low: 75<br>If too high: 30	If too low: 100<br>If too high: 25
**EM**	75	If too low: 100<br>If too high: 50	If too low: 125<br>If too high: 25
**UM**	125	If too low: 150	If too low: 175
